# Consultations

**Published:** 1877-05-20

**Authors:** 


CONSULTATIONS.—When a physician is called in consultation, he should be careful not to injure the medical brother in previous charge of the case. Consultations are seldom requested by the attending practitioner, because he feels the need of advice, though sometimes the conscientious medical man is actuated by this motive, and properly so, but rather to restore the waning confidence of the friends, and that they may know that the case is being judiciously treated. The physician consulted should, if possible, sanction what has been done, and, neither by direct nor indirect means, do anything which the friends can construe into a disparagement of the attending physician. Such conduct is in violation of the ethics, unworthy of the profession, and dastardly in principle.

